# Using electroencephalography to analyse drivers’ different cognitive workload characteristics based on on-road experiment

**DOI:** 10.3389/fpsyg.2023.1107176

**Published:** 2023-04-24

**Authors:** Ruiwei Liu, Shouming Qi, Siqi Hao, Guan Lian, Yeying Luo

**Affiliations:** ^1^Department of Naval Architecture and Marine Engineering, Guangzhou Maritime University, Guangzhou, China; ^2^School of Civil and Environmental Engineering, Harbin Institute of Technology Shenzhen, Shenzhen, Guangdong, China; ^3^Shenzhen Urban Public Safety and Technology Institute, Shenzhen, Guangdong, China; ^4^Department of Ports and Shipping Management, Guangzhou Maritime University, Guangzhou, China; ^5^School of Transportation and Architecture Engineering, Guilin University of Electronic Technology, Guilin, China

**Keywords:** EEG signals, cognitive workload, time-frequency transformation, EEG topography map, event-related spectral perturbation, inter-trial coherence

## Abstract

Driver’s cognitive workload has an important impact on driving safety. This paper carries out an on-road experiment to analyse the impact from three innovative aspects: significance analysis of electroencephalogram (EEG) under different cognitive workloads, distribution of EEG maps with different frequency signals and influence of different cognitive workloads on driving safety based on EEG. First, the EEG signals are processed and four frequencies of delta, theta, alpha and beta are obtained. Then, the time–frequency transform and power spectral density calculation are carried out by short-time Fourier to study the correlation of each frequency signal of different workload states, as well as the distribution pattern of the EEG topographic map. Finally, the time and space energy and phase changes in each cognitive task event are studied through event-related spectral perturbation and inter-trial coherence. Results show the difference between left and right brains, as well as the resource occupancy trends of the monitor, perception, visual and auditory channels in different driving conditions. Results also demonstrate that the increase in cognitive workloads will directly affect driving safety. Changes in cognitive workload have different effects on brain signals, and this paper can provide a theoretical basis for improving driving safety under different cognitive workloads. Mastering the EEG characteristics of signals can provide more targeted supervision and safety warnings for the driver.

## 1. Introduction

### 1.1. Background

The intelligence level of vehicles and transportation infrastructure has gradually improved, and the coordinated development of the transportation system amongst people–vehicle–road–environment will become the key to improving road safety (Fu and Sayed, 2021). From the perspective of the driver, how to obtain the driver’s status and response information is the focus of current research on driving behaviour. The drivers require not only the coordination of body movements but also the cognition of the brain to coordinate control and command completion. Studying the correlation mechanism between the brain and driving behaviour will reveal the internal connection of the driver’s physiological and behavioural responses in driving activities from the perspective of the interdisciplinary aspects of neuroscience and human factors and will thus be applied to new technologies such as vehicle driving assistance systems, resulting in strong practical significance.

Driven by research programs around the world, brain science research on human attention and cognition is gradually developing. There are many research methods in cognitive science, and electroencephalogram (EEG) signal analysis is the most common one. EEG signals are changes in the scalp surface points caused by the activity of brain neurons, reflecting the functional state of the brain and extracting effective EEG information; detecting changes in these potentials can provide a deeper understanding of the brain’s activity function (Jung et al., 1997). The mental workload is the result of the interaction amongst work motivation, task demand, cognition and behaviour when people complete tasks (Moray, 1979). When the task demand is higher, more mental workload is usually required (Eduardo and Dan, 2010). The driver will be distracted by many distractions during driving, and including cognitive distraction will affect the driver’s mental workload.

### 1.2. Literature review

Distracted driving caused by secondary tasks has been identified as a major threat in driving, causing serious fatal crashes. Secondary tasks are often used in the context of driving to measure the attentional demands of the primary task of driving (Huemer et al., 2018). Secondary tasks include technics-related secondary behaviours (such as using smartphones, manipulating dashboard controls, using a satellite navigation device and wearing headphones or earbuds) and non-technical distraction behaviours (such as eating, drinking, smoking, talking and grasping for objects; Motamedi and Wang, 2016; Arvin and Khattak, 2020). The cognitive workload will have a greater effect on the driver’s attention distribution, transfer, reaction speed and short-term memory, which will reduce driving performance and thus affect driving safety. Many scholars have researched cognitive tasks. Common cognitive workload tasks include N-back, math problem calculation and Stroop test. Through the different signal collections, EEG showed its superior utility in studying human cognitive workload. Hogervorst et al. (2014) completed the N-back tasks of varying degrees by recording EEG, electrocardiographic and eye movement indicators. EEG was found to have the best effect and could better show the relationship between physiological variables and mental workload. To study the task workload estimation of EEG data, the subjects of multi-attribute task EEG data cross-validation and 11 types of calculation methods were compared by Christian et al. The results showed that EEG-based workload classification requires multiple frequencies; compared with the other 10 cognitive state assessment methods, EEG data has superiority in frequency bands (Christian and Scott, 2011). Muhl et al. (2014) used the time-domain and the frequency-domain analysis to classify different workloads by designing two different workload tasks and recording EEG signals and other physiological states. To study the influence of different cognitive tasks (mathematical and decision-making problems) on the driver’s cognitive state, Almahasneh et al. (2014) designed distracted driving and simple driving in the driving simulation process. The results showed that different characteristics of the secondary tasks have different effects on EEG response, and the location in the frontal lobe was different. The most affected area during distracted driving is the right frontal lobe.

Brain signals are distributed in different brain regions, potentially directly reflecting neural signals of brain activity and are highly correlated with the driver’s real-time state (Thammasan et al., 2017). Many scholars have studied the effect of EEG on drivers’ cognitive distraction workload by extracting the characteristic quantities of brain waves for data analysis. To study the relationship between distraction and EEG during driving, Lin et al. (2011) designed a dual-task test including math tasks using a simulation-based driving study. The results showed that the increase in the power of the theta and beta bands is related to the distraction effect of the frontal lobe of the brain. In the case of driver attention distraction, the increase of power in the frontal area is related to the driver’s distraction. Yang et al. (2018) studied the relationship between driving behaviour and brain activity through driving simulation experiments, used power spectrum analysis to process EEG signals and used Pearson correlation analysis for statistical analysis. The results showed that normal driving behaviour is associated with the four areas of the brain, especially the temporal, occipital and frontal regions; β log-transformed power is most relevant to ordinary driving behaviour. Wang S. et al. (2015) predicted the starting and ending moments of distracted driving through the EEG driving simulation test and detected brain activity through EEG signals to determine the driver’s distraction. By studying the relationship between EEG signals and driving fatigue, Jap et al. (2009) found that with the deepening of fatigue, the alpha wave increased significantly and the beta wave decreased significantly. Lei and Roetting (2011) studied the relationship between EEG and the driver’s mental state by adding three task workload conditions to the driver through driving simulation tests, finding that with the increase in workload, alpha and theta activities increased significantly and were very sensitive.

From the perspective of current studies, great progress has been made in studying the driver’s cognitive workload from EEG signals. However, the experiment environment is mainly dominated by driving simulation, and the method of data analysis has not changed much. From the point of view of brain electrical signal processing, given that the interference task of the experimental design is known, the composition of the electrical activity associated with the interference event has become the focus of research on cognitive workload. In the current research, studies on different cognitive workloads are lacking, and most studies have focused on the effects of different waves, with less attention to the effects of different brain regions.

In this paper, an on-road driving test was conducted, and the data were collected in the normal driving state and the different levels of cognitive workloads. The time-frequency distribution of different EEG signals generated by the left and right brain was studied, as was the distribution of different frequencies in different brain regions in different cognitive workloads. The distribution and energy characteristics under different cognitive events were studied to explore the regular pattern of EEG characteristics of drivers’ brains under different cognitive workloads, as well as the changing trend of monitor, perception, visual and auditory channels in different driving states.

## 2. Experimental design

### 2.1. Participants

Many studies have proved that small samples can also be used in driver characteristic tests. Usually, the number of samples should be greater than six to be effective (Ahlstrom and Kircher, 2017; Biswas and Prabhakar, 2018; Stapel et al., 2019). A total of 20 individuals (10 males and 10 females) with a mean age of 34 years old (SD = 8.65, age distribution ranges from 25 to 55 years) were recruited from the university community, they have a mean driving experience of 7 years (SD = 6.96). The purpose was to select a group of people who were representatives of the drivers in the urban traffic environment. For driving safety, they need to be familiar with the local traffic environment and obtained driving licenses. According to the preliminary screening survey, they had 20/20 or corrected-to-normal vision with no mobility impairments. They were not allowed to drink or smoke the day before the experiment and remained healthy.

### 2.2. Apparatus and experimental routes

One conventional vehicle (2017 SAGITAR 1.6T) was used, with a human driver controlling the throttle, brake and steering. To capture data related to the driver’s actions and status in real time, the vehicle was equipped with a laser ranging sensor (ranging range of 0.5–200 m, ranging accuracy of ±0.5–1 m, measuring frequency of 10–50 Hz), radar rangefinder and four synchronised cameras (recording the front and rear of the vehicle, the driver’s face and on-board operation video). The driver wore an EEG collection device (NeuroOne EEG, with 21 sampling channels) during driving. The drivers operated in accordance with their own daily driving behaviours, thereby collecting their natural driving behaviour in a real environment.

Non-peak times on working days were chosen for the times for the road test (9:30–11:00 a.m., 2:30–4:00 p.m.). The weather was clear and the temperature was suitable for driving. All the testers conducted real-drive tests on the same road section to ensure that all drivers’ traffic conditions, sunshine conditions, weather and other factors during the test were similar. The real car test was selected to be at Zhongyuan Avenue, Harbin City. The starting point is the intersection of Songpu Bridge and Zhongyuan Avenue. The endpoint is the intersection of Zhongyuan Avenue and Xiang’an North Street. The whole length of the test road section is 10 km, and the test road is a two-way six-lane road separated by the central separation zone. The traffic flow in this section is small and stable, with less traffic interference, good road alignment, good driver’s vision and high safety for cognitive road tests.

**Figure 1:**
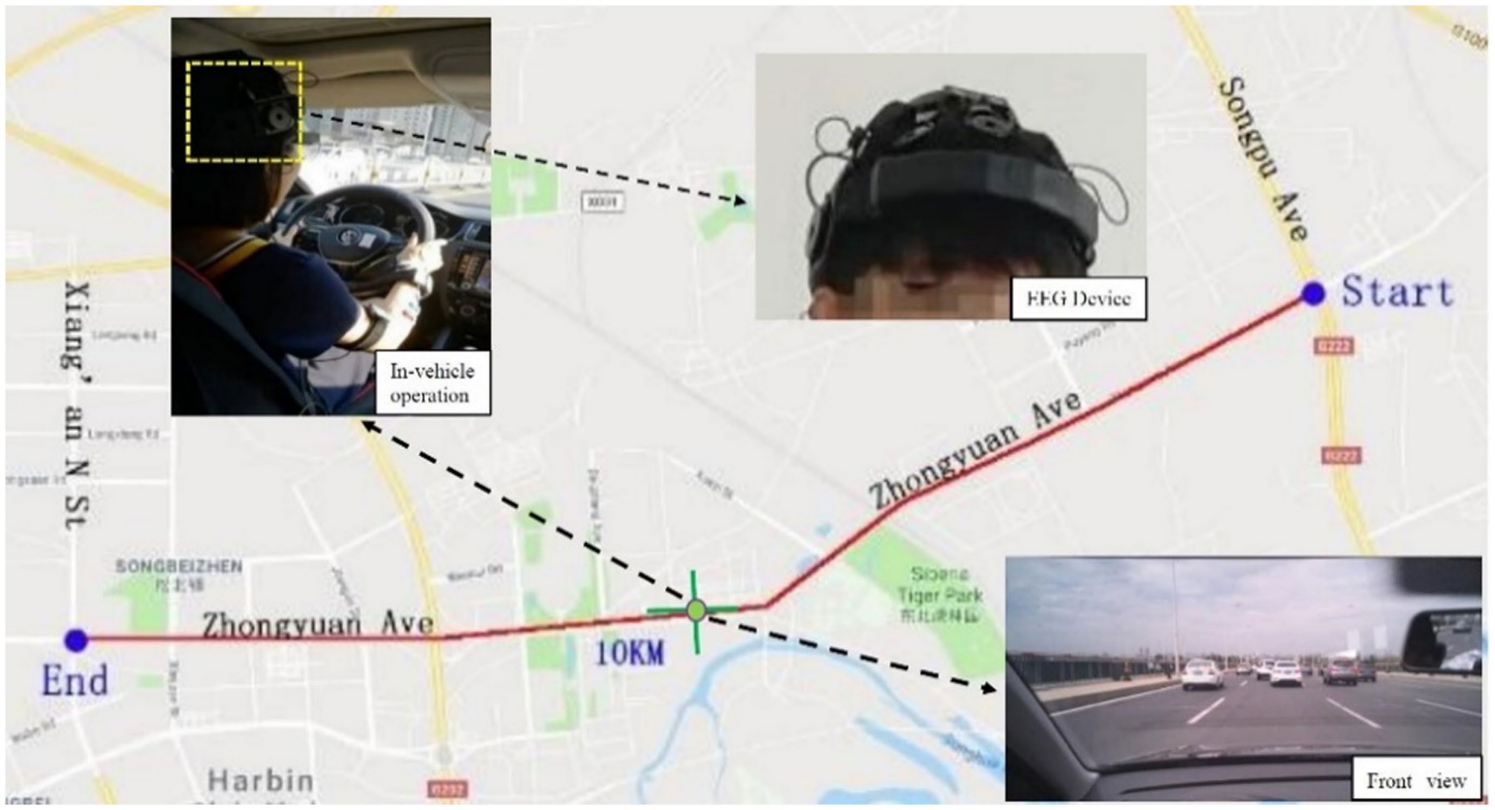
Experiment routes and the traffic environment.

The experimental route, traffic environment, and the EEG device worn by the drivers are shown in Figure 1.

### 2.3. Experimental process

1. The staff introduced the driving test process to the participants and asked for their personal information. After confirming that participants are qualified, the staff assisted the participants in wearing the device.
2. The staff guided the driver to the test vehicle, turned on all the test equipment and completed the calibration and synchronisation of the equipment. In addition to the participant, two staff members were in the vehicle: one was responsible for the adjustment of the experimental equipment, and the other was responsible for issuing the instructions required for the experiment.
3. Once the preparations were ready, participants were allowed to drive in a safe area for ~15 min to accommodate the vehicle and the collection device.
4. Participants drove into the designated test route and started driving. The staff marked the starting point and began to record data. Unless there was a safety hazard, participants should cooperate with the instructions required to complete the experiment under the guidance of staff.
5. On certain road sections, the driver performed cognitive workload tasks while maintaining the main driving tasks. They completed the corresponding mathematical calculations according to the experimental design settings. In the event of a dangerous situation, the driver had the right to choose to terminate the task.
6. After reaching the ending point, the staff marked the camera and ended the recording of the experimental instruments. After turning off all the instruments, they led the driver to a designated place to rest and removed the device for him/her.
7. Participants completed the final questionnaire and received a test reward (CNY 200). Staff copied and archived the test data.

In the setting of tasks, from the initial distraction such as language communication and phone calls to the cognitive tasks with memory ability such as N-back and mathematical calculation (Wang Y. K. et al., 2015), this experiment adopts listening and calculating mathematical problems. All the participants have a good mathematical foundation. The difficulty level of the problem is divided into three levels: simple, general and complex, in which drivers produce mild, moderate and deep cognitive workloads, respectively, when completing distracted tasks of different difficulty levels. The research group conducted many experiments to improve and verify the calculation difficulty classification in the early stage, including the number of questions at each level, as well as the time for thinking and answering. Five simple tasks were given, comprising two types: addition calculation of two-digit positive numbers without carrying (such as 21 + 23) and subtraction calculation of two-digit positive numbers without borrowing (such as 25–12), and the time given for each question was 6 s. Three general tasks were given, comprising two types: addition calculation of two-digit positive numbers with carrying (such as 16 + 17) and subtraction calculation of two-digit positive numbers with borrowing (such as 54–18), and the time given for each question was 10 s. One subtraction calculation of two-digit negative numbers (such as 18–33) and one continuous addition calculation of two-digit positive numbers (such as 12 + 23 + 36) were included in complex tasks, and the time given for each question was 15 s. Each stage takes about 30 s and the total time of the test is about 90 s, with each participant needing to complete the experiments twice.

This paper assumes that in the cognitive workload on-road experiment, the driver’s cognitive workload is completely generated by calculating mathematical problems of different difficulty and has nothing to do with the traffic environment.

## 3. Methodology

### 3.1. Data processing

**Figure 2:**
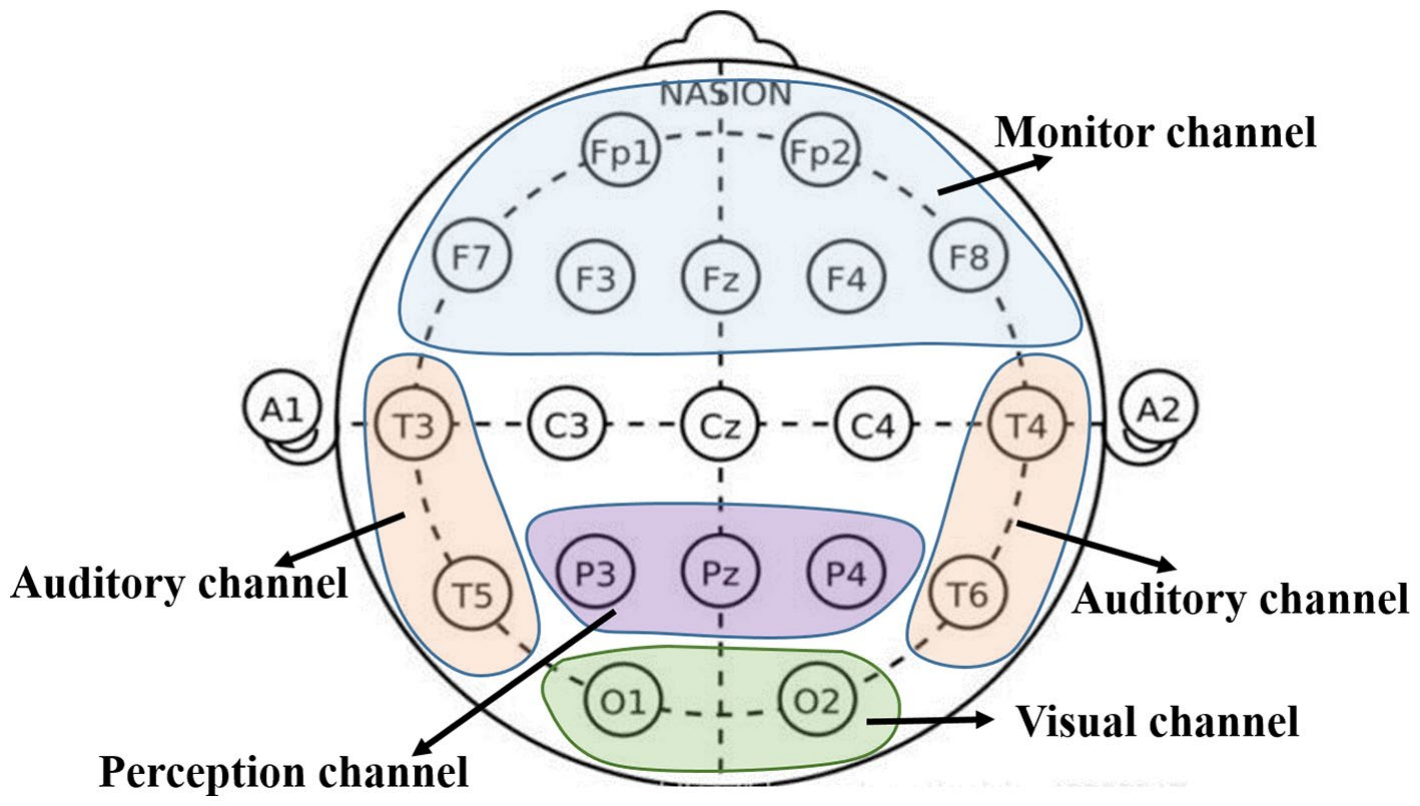
Electrode position and different functional channels diagram.

All EEG signals were collected from 19 scalp points and binaural electrodes. The brain regions have different functions, amongst which the frontal area is the monitor centre, the parietal area is the perception centre, the occipital area is the visual centre and the temporal area is the auditory centre (Euler, 1736; Finn et al., 2014). The schematic diagram of electrode positions and different functional channels is shown in Figure 2, and the distribution of electrode positions in the brain area is shown in Table 1.

**Table 1:** Electrode position distribution.

Location	Electrode name		
	Left	Right	Central
Prefrontal Lobe	Fp_1_.	Fp_2_	—
Inferior Lobe	F_7_	F_8_	—
Frontal Lobe	F_3_	F_4_	F_Z_
Central Lobe	C_3_	C_4_	C_Z_
Temporal Lobe	T_3_	T_4_	—
Posterior Lobe	T_5_	T_6_	—
Parietal Lobe	P_3_	P_4_	P_Z_
Occipital Lobe	O_1_	O_2_	—
Auricular	A_1_	A_2_	—

The signal was sampled by a 50 Hz notch filter at a sampling frequency of 256 Hz, and all electrodes were referenced by the average electrode. To obtain higher confidence in the data, the original EEG signal was divided into 5 s non-overlapping windows, and the useless electrodes were removed according to the sample (Croft and Barry, 2000). Then, all the data were re-referenced. In checking the data, if the data of a channel was broken, it could be corrected by interpolation. Ultimately, 43,200 pieces of data were sorted out.

In the process of collecting the driver’s EEG data, there will be a variety of interference sources, such as electrooculogram (EOG), electromyography and electrocardiogram, resulting in overlapping frequency band information. EOG is a general term for electrical signals caused by the left and right movement of the eye and blinking. EOG can be divided into three types: vertical (VEOG), horizontal (HEOG) and radial (REOG). The amplitude and frequency of these three types of EOGs are different. Comparatively, the amplitude of VEOG noise caused by blinking is the strongest and has the greatest impact on the signal (Brunia et al., 1989). Compared with EOG noise, EMG and ECG noise have lower amplitudes and lower probability of occurrence. Generally, they can be directly filtered out during signal processing. Therefore, the removal of EOG signals is also the key to data processing (Wim de Clercq et al., 2006).

**Figure 3:**
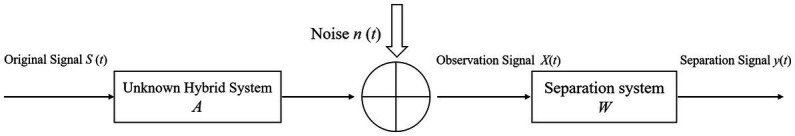
Schematic diagram of the ICA principle framework.

In this paper, independent component analysis (ICA) was used to remove electroocular interference. ICA is a method of blind source separation, which can separate the original signals of multiple hidden signal sources from the mixed signals of the signals simultaneously emitted by the multi-dimensional signal sources (Hyvarnien, 1999). Based on the essence that the signals emitted by multiple signal sources belong to a non-Gaussian distribution and are independent of each other, under the condition that the source signal and the mixing matrix are unknown, a group of random variables can be represented as a linear combination of statistically independent variables. This method can minimise the statistical dependence between the components of the analysed signal and highlight the essential results of the source signal. The basic principle is shown in Figure 3.

ICA includes two processes of mixing and unmixing, as follows (Hyvarinen et al., 2002; Efimov, 2014):

There are *n* times independent signal sources $S=\left(S_{1},S_{2},\ldots ,S_{n}\right)$ ; each dimension is a random signal, which is independent of each other. A is an unknown mixing matrix, *a_i_* is the base vector of the mixing matrix *A*, used to combine the superimposed signal *S*, *X* is an observation matrix, which is composed of an *n*-dimensional observation vector $X=\left(x_{1},x_{2},\ldots ,x_{n}\right)$ . Then, there is


(1) $$X=AS=\overset{n}{\underset{i=1}{\sum }}aisj,i=1,2,\cdots n$$

The purpose of ICA is to find a linear transformation matrix (separation matrix) *W* of the mixed observation signal *X*, so that the output is as independent as possible, namely:


(2) y(t)=WX=WAS

Given that the mixed matrix *A* is an unknown matrix, the independent source signal is also a hidden variable that cannot be directly observed. The only variable that can be observed is the random variable *X*. If no other conditions apply, the solution of Equation (1) must be multiple solutions. ICA needs to go through the unmixing model and give the unique solution of the equation to realise the extraction of independent components. Separation matrix *W* = (*w_ij_*) has been constructed. After the transformation of *X* through the separation matrix *W*, the *n*-dimensional output column vector is obtained, and its unmixing model can be expressed as


(3) Y=WX=WAS=GS

where *G* is the system matrix. If *G* = *I* (*I* is the identity matrix of *n* * *n*) through learning, then *y* = *s*, thus achieving the goal of separating source signals.

The ICA algorithm is used to estimate the coefficient matrix *W* to obtain the raw signal. After running ICA and the signal removing the artifact, analysing the relative influence of the human brain’s electrical activity on each electrodeposition is possible.

**Figure 4:**
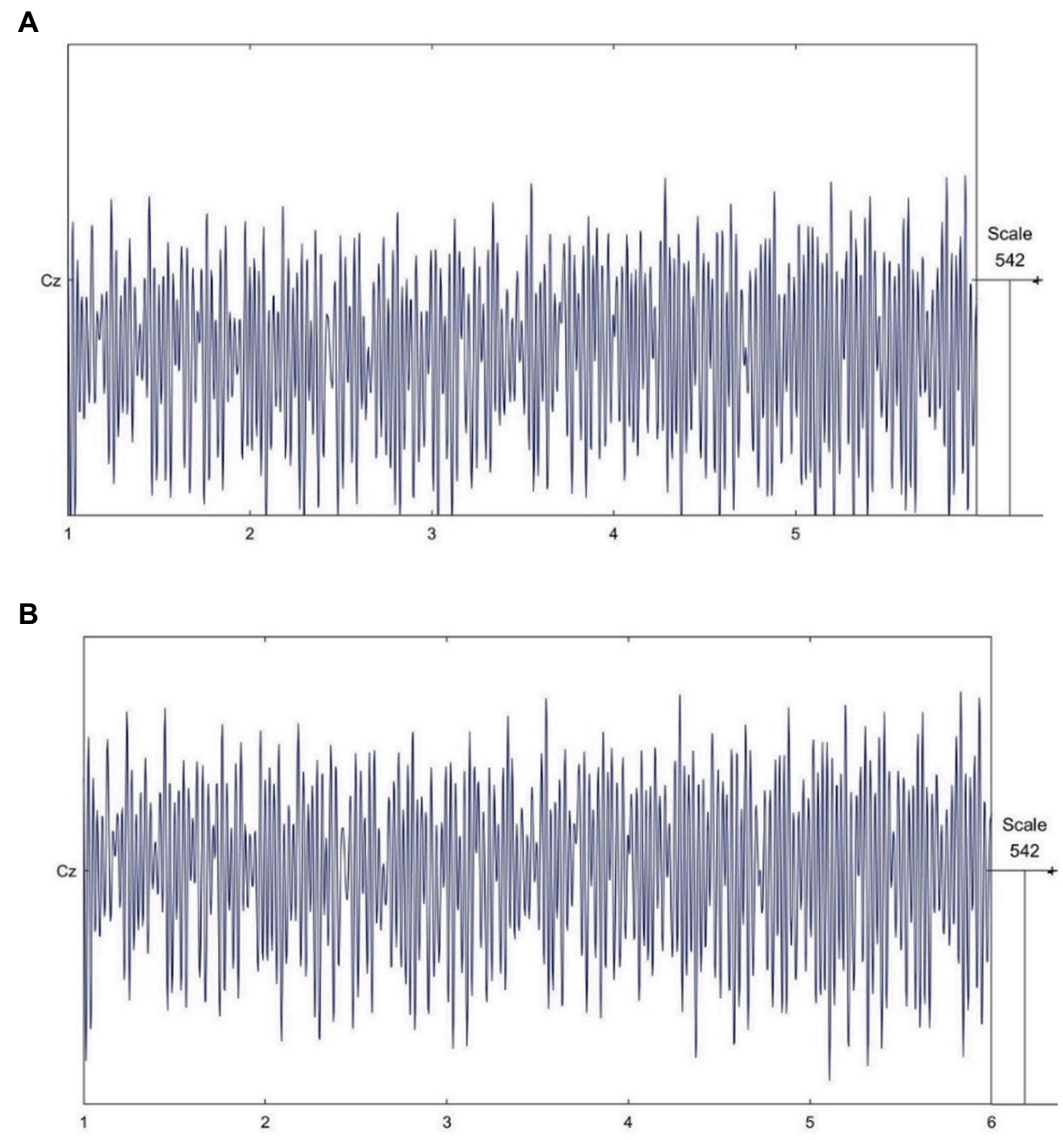
EEG signal data process. **(A)**. Raw EEG signals. **(B)**. EEG signal after removing electrooculogram artifacts by ICA.

Taking the *C_Z_* channel as an example, the raw EEG signal is shown in Figure 4A, and the signal after removing the EOG artifacts by ICA is shown in Figure 4B; the original data can be optimised after this process.

### 3.2. Analysis of energy and phase characteristics based on time-frequency analysis

#### 3.2.1. Short-time Fourier transform

For the extraction of EEG time-frequency features, this paper uses the short-time Fourier method, proposed by Dennis (1946), which can be used to solve the frequency and phase of the sine wave of the variable signal in the progress area. The expression is


(4) $$STFT\left(t,w\right)=\int_{-\infty }^{+\infty }x\left(\tau \right)w^{*}\left(\tau -t\right)e^{-jwt}d\tau$$

where *w* is the angular frequency, and $w^{*}\left(\tau -t\right)$ is the complex conjugate function to $w^{*}\left(\tau -t\right)$ . *w*(*t*) is the selected window function, in which the rectangular window, triangular window, and Hamming window can usually be used. The selection can be made according to the needs when using, potentially improving resolution and reducing spectrum leakage. In this study, we chose to use the Hamming window, which can be expressed as


(5) $$w\left(t\right)=0.5\left(1-\cos\frac{2\pi t}{T}\right),0\leq t\leq T$$

#### 3.2.2. Power spectral density estimation

The power spectrum is a method with a window function to reveal the power change of the EEG signal through the Welch method (Welch, 1967), which is an improvement of the periodic graph method (Yu et al., 2018). For the *N* points of the time series *x*(*t*), it can be divided into *N* segments, and the time length of each segment is *M*. Then, the power spectrum of Welch is estimated as


(6) $${\tilde{B}}_{x}\left(w\right)=\frac{1}{p}\overset{p}{\underset{m=1}{\sum }}j_{m}\left(w\right)$$

where *j_m_*(*w*) is a modified period diagram of segment *M*, which can be expressed as


(7) $$j_{m}\left(w\right)=\frac{1}{MU\left(w\right)}\overset{M-1}{\underset{t=0}{\sum }}{\left|x_{m}\left(t\right)w\left(t\right)e^{-jwt}\right|}^{2}$$

where *x_m_*(*t*) is the time series of the *m*-th segment.

*U*(*w*) is a normalisation factor, which guarantees that the resulting power spectrum is progressive and unbiased, which can be expressed as


(8) $$U\left(w\right)=\frac{1}{M}\overset{M-1}{\underset{t=0}{\sum }}w^{2}\left(t\right)$$

#### 3.2.3. Event-related spectral perturbation and inter-trial coherence feature extraction

For cognitive EEG signals, it is usually necessary to observe the energy and phase changes corresponding to time and space in each cognitive task event and lock the power spectrum of the EEG segment to the same event in the time domain. Then, the average power change is calculated according to the frequency; a two-dimensional time-frequency image with event-related spectral perturbation (ERSP) can be obtained. First, the short-trial Fourier transform is performed on the single trial, and then the ERSP and the inter-trial coherence (ITC) are superimposed between the trials based on the obtained time-frequency value (Ming et al., 2010; Ahirwal et al., 2014). ERSP characterises the energy change of a single test over time and reflects the influence of the cognitive process on the power spectrum of each frequency band. ITC represents the feature of the degree of phase-locking change with time. It shows the degree of phase synchronisation relative to a set of test events at a specific waiting time and frequency (Tallon-Baudry et al., 1996).

For *n* times cognitive events, the ERSP is defined as


(9) $$ERSP\left(f,t\right)=\frac{1}{n}\sum {\left|F_{k}\left(f,t\right)\right|}^{2}$$

where *F_k_* (*f*,*t*) is the time-frequency distribution of cognitive EEG for the *k*-th event.

ITC is defined as


(10) $$ITC\left(f,t\right)=\frac{1}{n}\left|\overset{n}{\underset{k=1}{\sum }}\frac{F_{k}\left(f,t\right)}{\left|f_{k}\left(f,t\right)\right|}\right|$$

The non-coupling range of the ITC value is between 0 and 1. If ITC = 1, this component is phase-locked, indicating that the cognitive electroencephalogram is strictly locked to the initial state of stimulation of the cognitive event; if ITC = 0, it means that this component is not phase-locked.

## 4. Results and discussion

### 4.1. Correlation of different frequency signals in different cognitive workloads

The left hemisphere controls people’s action behaviours, such as language and calculations, while the right hemisphere controls people’s imagination, spatial thinking and intuitive feelings. Therefore, studying the left and right brains separately is necessary. This paper extracts the amplitudes of the driver’s delta, theta, alpha and beta EEG signals under the normal driving state and different levels of cognitive tasks, and the paired *t*-test of the sample was performed. The data of the left and right cerebral hemispheres were analysed, and the results are shown in Table 2.

**Table 2:** Value of *p* and *t*-value from Pair-sample *T*-test of all frequency bands under normal driving and different cognitive workloads.

Test pair	*p*-value (L)	*t*-value (L)	*p*-value (R)	*t*-value (R)
Delta_Normal—Delta_Mild	0.032*	−2.313	0.036*	−2.302
Delta_Normal—Delta_Moderate	<0.001***	−4.879	0.022*	−2.565
Delta_Normal—Delta_Deep	0.004**	−3.276	0.032*	−2.361
Delta_Mild—Delta_Moderate	0.038*	−2.233	No Sig	—
Delta_Mild—Delta_Deep	No Sig.	—	No Sig	—
Delta_Moderate—Delta_Deep	No Sig.	—	No Sig	—
Theta_Normal—Theta_Mild	No Sig.	—	0.034*	−2.337
Theta_Normal—Theta_Moderate	<0.001***	−4.353	0.003**	−3.549
Theta_Normal—Theta_Deep	0.002**	−3.664	0.001**	−4.083
Theta_Mild—Theta_Moderate	No Sig.	—	No Sig	—
Theta_Mild—Theta_Deep	No Sig.	—	0.033*	−2.350
Theta_Moderate—Theta_Deep	No Sig.	—	0.044*	−2.202
Alpha_Normal—Alpha_Mild	0.032*	−2.308	<0.001***	−4.865
Alpha_Normal—Alpha_Moderate	No Sig.	—	<0.001***	−4.677
Alpha_Normal—Alpha_Deep	0.033*	−2.299	0.003**	−3.574
Alpha_Mild—Alpha_Moderate	No Sig.	—	No Sig	—
Alpha_Mild—Alpha_Deep	No Sig.	—	No Sig	—
Alpha_Moderate—Alpha_Deep	No Sig.	—	No Sig	—
Beta_Normal—Beta_Mild	<0.001***	−4.253	<0.001***	−4.973
Beta_Normal—Beta_Moderate	<0.001***	−4.943	0.001**	−4.056
Beta_Normal—Beta_Deep	<0.001***	−5.767	<0.001***	−5.356
Beta_Mild—Beta_Moderate	No Sig.	—	No Sig	—
Beta_Mild—Beta_Deep	0.006**	−3.130	0.009**	−3.013
Beta_Moderate—Beta_Deep	0.016*	−2.633	No Sig	—

*p < 0.05; **p < 0.01, ***p < 0.001, L stands for left brain hemisphere, and R stands for right brain hemisphere.

Analysing the data shows that after the cognitive task, the driver’s brain activity has different changes, and the different frequencies are different in different brain regions. Results showed significant changes in the electrical activity of the left and right hemispheres of the brain, highlighting the influence of different cognitive workloads.

From the delta wave data, we can see that driving under mild, moderate and deep cognitive workloads is more significant than the normal driving state (driving without cognitive tasks) on both sides. Amongst these workloads, in the left hemisphere, the moderate cognitive workload is the most significant (*t*-value = −4.879, three-level significance). Next is deep cognitive workload (*t*-value = −3.276, second-level significance), and mild and moderate cognitive workload also directly have first-level significance. On the right side of the brain, different cognitive tasks are similar to the normal driving state in significance, and all have first-level significance. The cognitive workload of different degrees has no significance directly. According to the comprehensive analysis results, the delta wave has a greater influence on the left brain than the right brain under the effect of workload.

Theta wave data show that in the left brain, only moderate and deep cognitive workload will have a significant impact on a normal driving state, and moderate cognitive tasks will produce three-level significance compared with normal driving (*t*-value = −4.353). As for the right brain, different cognitive states have a significant effect on the normal driving state, and deep cognitive workload has the most significant impact on the normal driving state (*t*-value = −4.083, second-level significance). Deep cognitive workload also has a first-level significant effect on both mild and moderate cognitive workloads.

The alpha wave shows obviously different effects on the left and right brain. In the left brain, only mild and deep cognitive workloads have first-level significance for normal driving conditions. In the right brain, all three different degrees of cognitive workload have a significant impact on the normal driving state, and mild and moderate cognitive workloads have a three-level significant effect on the normal driving state (*t*-value were −4.865 and −4.677, respectively). No significant effect on the left and right brain between different degrees of cognitive workload was found.

Beta waves have the greatest impact on normal driving states and driving under different levels of cognitive distraction tasks. On the left side of the brain, the three levels of cognitive distraction workload all have a three-level significant impact on the normal driving state. As the cognitive distraction workload increases, the significant impact intensifies, and the significant degree of deep cognitive workload on normal driving was the largest (*t*-value = −5.767). Deep cognitive distraction also had secondary and first-order significant effects on mild and moderate cognitive distraction, respectively. In terms of the right brain, three levels of cognitive distraction workload also have a strong and significant effect on normal driving, with the greatest effect on depth (*t*-value = −5.356), followed by mild (*t*-value = −4.973). Deep cognitive tasks also produce a second-level significance for mild cognitive workloads (*t*-value = −3.013).

### 4.2. Distribution of EEG topographic maps with different frequency signals

**Figure 5:**
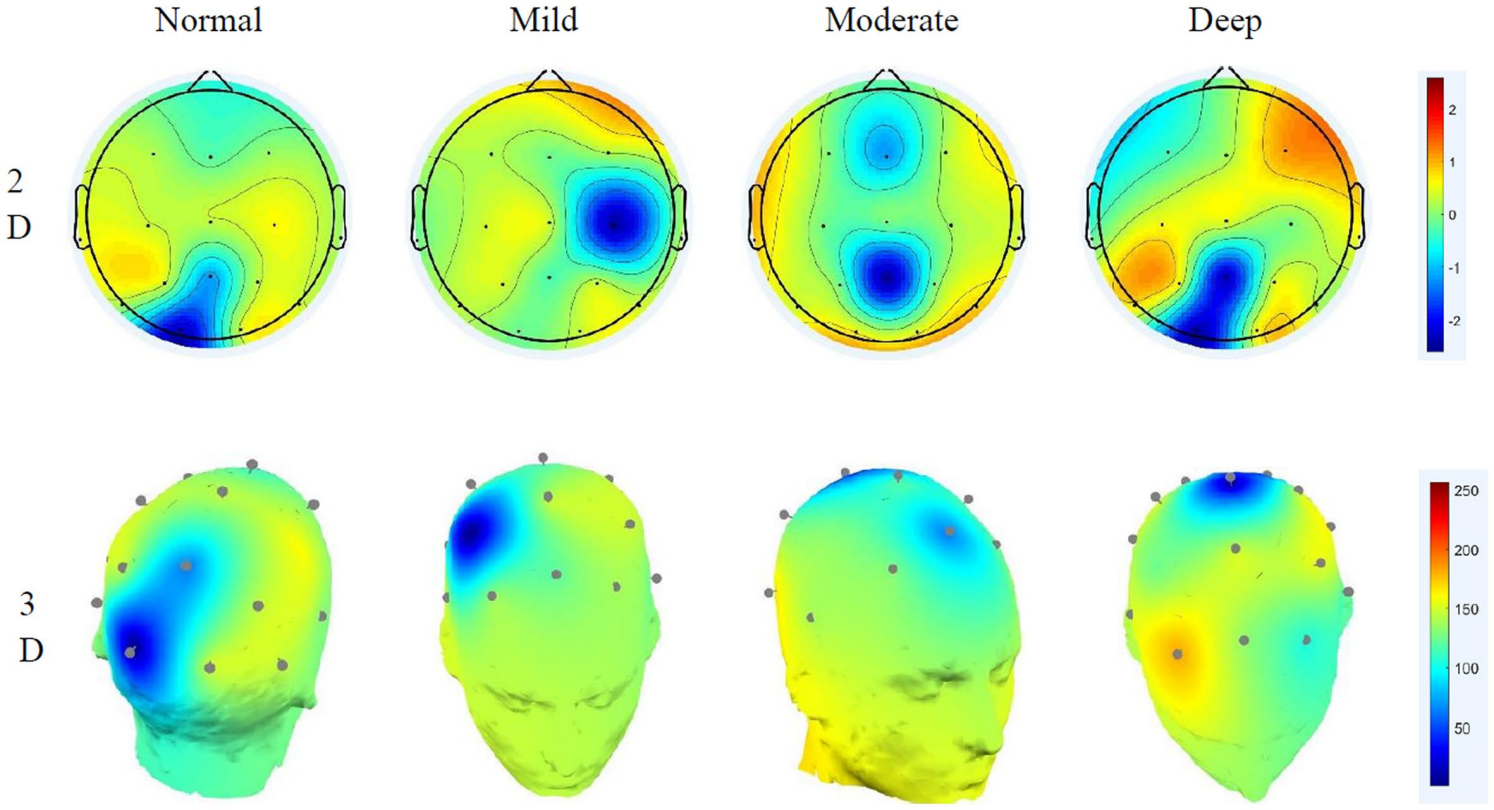
Delta wave 2D–3D EEG topography in different states.

**Figure 6:**
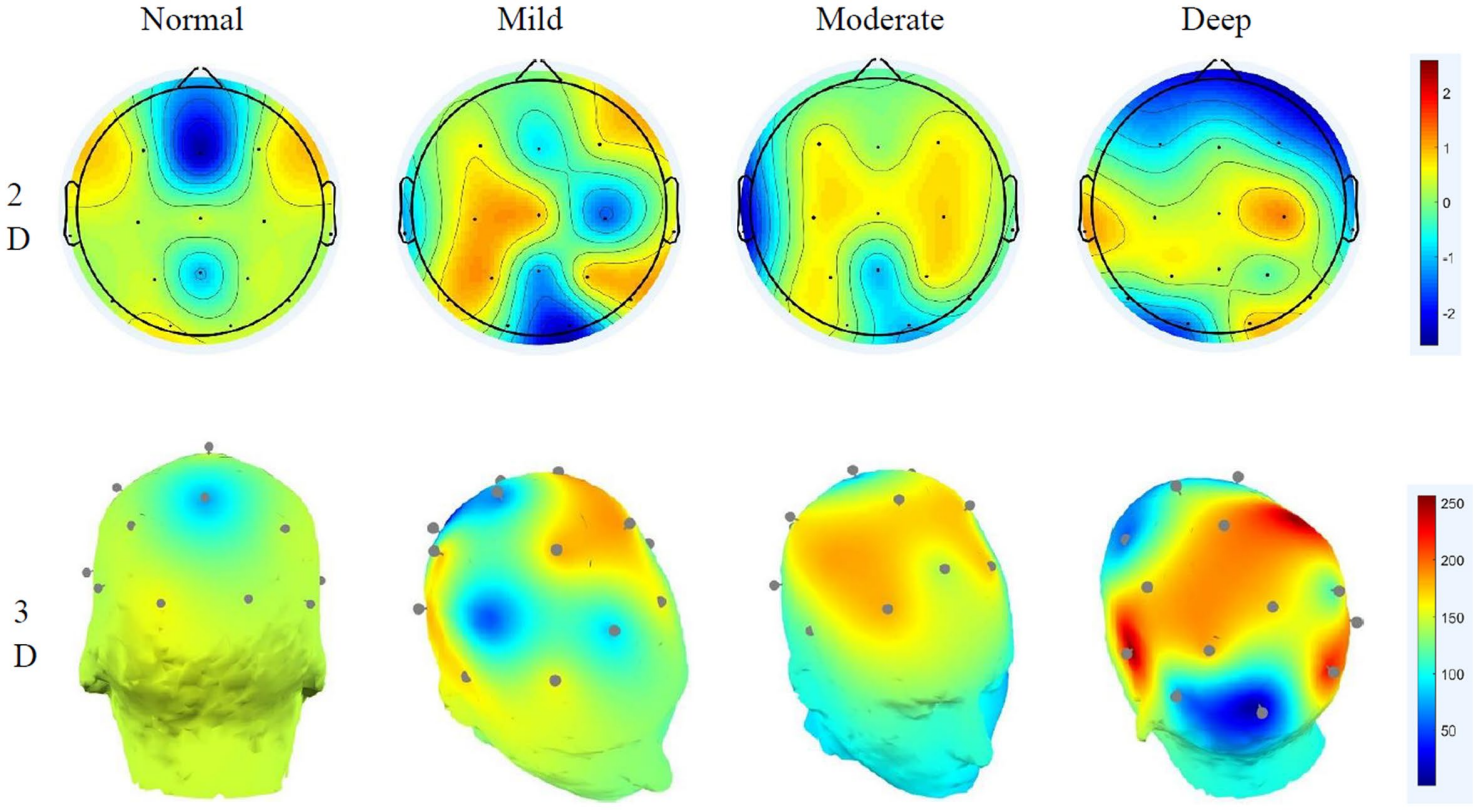
Theta wave 2D–3D EEG topography in different states.

**Figure 7:**
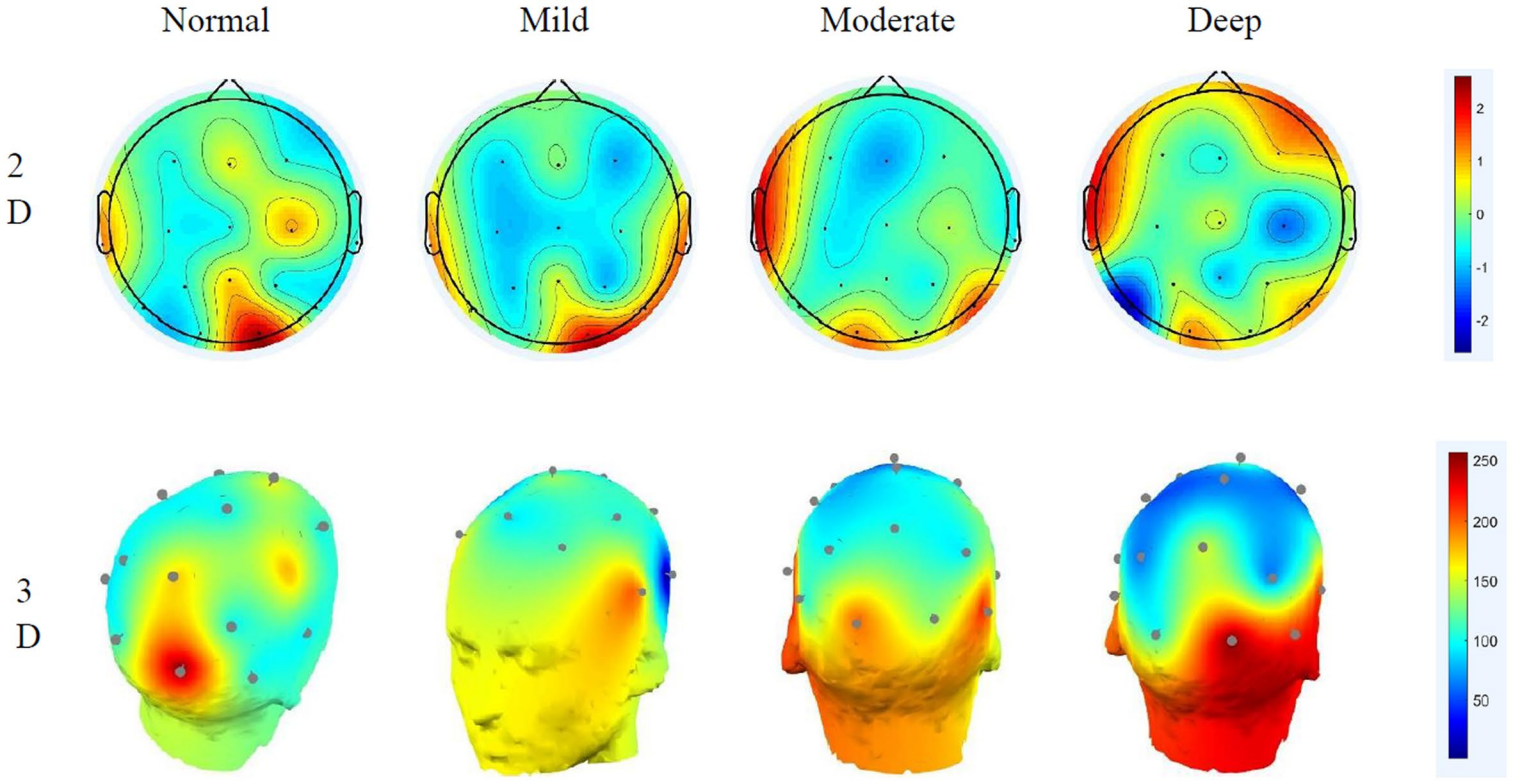
Alpha wave 2D–3D EEG topography in different states.

**Figure 8:**
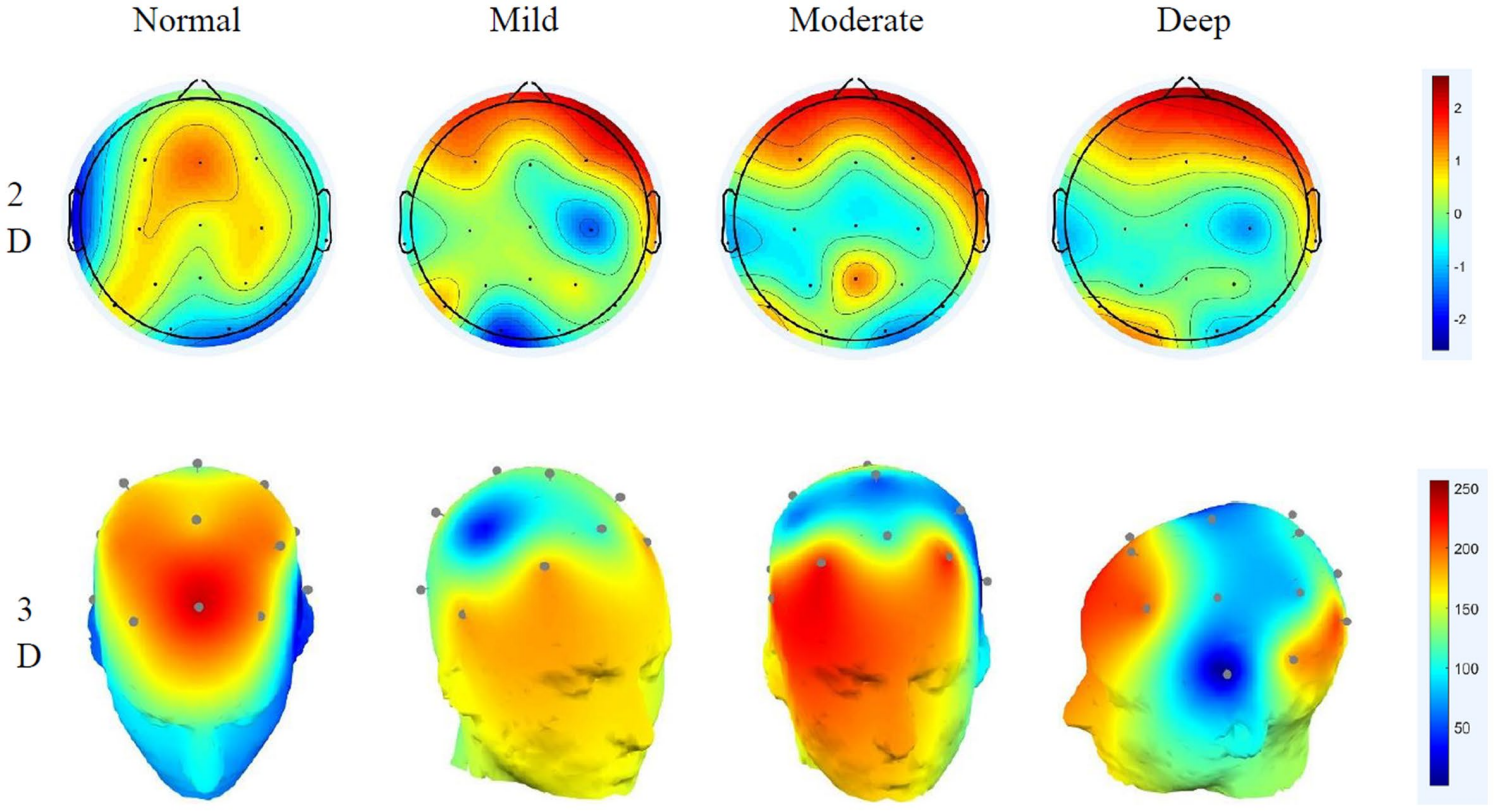
Beta wave 2D–3D EEG topography in different states.

The paired t-test of the sample can only obtain the significance of different cognitive workloads to the normal driving state and cannot see the specific area of brain activity, which requires EEG topographic map analysis (Chiarelli et al., 2010). Through short-time Fourier and power spectrum estimation, the brain topographic maps of the affected areas of delta, theta, alpha and beta in the brain in different states are obtained, as shown in Figures 5–8.

#### 4.2.1. Delta wave EEG distribution characteristics

The characteristic of the delta wave is that it is not controlled by nerves in low-level parts. Comparing the topographic maps of the delta wave in different states shows that under normal driving conditions, the brain is more evenly distributed and the activity intensity is weak. Under the effect of mild cognitive workload, the activity in the frontal region is strengthened. Until deep cognitive workload, the activity of the frontal and temporal lobe increases slightly, and the activity of the parietal lobe gradually weakens. The temporal lobe mainly affects human hearing and memory, indicating that delta waves are related to human memory. The 3D graph shows that owing to the low frequency of delta, the energy of its overall activity is not large, and its influence during the entire process of cognitive workload changes is minimal.

#### 4.2.2. Theta wave EEG distribution characteristics

Theta waves are typically enhanced when people are disappointed or frustrated. Comparing the theta brain topography maps in different states shows that in the normal driving state, the activity in the central parietal lobe is weaker, and the energy in the temporal part is slightly stronger than in other brain regions. As the cognitive workload increases, the activity in the frontal region began to strengthen significantly, and the activity in the temporal area also gradually increased. The frontal lobe is closely related to the mathematics and logic of the brain. The increase in frontal activity is precisely due to the increased difficulty of cognitive workload math problems. The calculations of the brain are closely related, and, at the same time, affect judgement. The 3D EEG topographic map that the energy change of the theta wave is more obvious under different driving workloads.

#### 4.2.3. Alpha wave EEG distribution characteristics

Alpha waves are generally the most common type of wave in humans. They are characterised by a decrease in amplitude when human attention is more concentrated and an increase in amplitude when taking a deep breath. Considerable research has shown that when performing different cognitive workload tasks, the activation energy and distribution are more obvious (Klimesch, 1999), and the results of this study also verify this idea. Under normal driving conditions, occipital activity is relatively strong. Under the effect of mild cognitive workload, the activation energy of the temporal region begins to increase. This is caused by the calculation of math problems occupying the driver’s memory space. With the aggravation of cognitive workload, the activity at the occipital region is significantly enhanced, while the activity at the frontal is obviously reduced, indicating that the deepening cognitive workload will affect the driver’s visual function. The 3D EEG topographic map clearly shows that under different conditions, alpha’s energy has a large range of changes, its rhythm enhancement activity is obvious, and it has a greater impact on the right brain. The occipital is closely related to the driver’s cognitive executive ability, which affects the driver’s behaviour monitoring ability (Lorist et al., 2005). Analysis shows that the alpha wave is related to the human visual and behaviour monitoring ability, and the deeper the distraction load is, the lower the driver’s behavioural monitoring ability will gradually be.

#### 4.2.4. Beta wave EEG distribution characteristics

The beta wave is the most frequent type of wave. Generally, when people are in a state of tension or emotional instability, its amplitude will increase significantly. In the normal driving state, beta waves mainly appear in the parietal and temporal lobes, and the activation energy is relatively strong. Under the effect of distraction, the activity in the frontal area is significantly increased. Under the deep cognitive workload, relatively strong energy was observed in the occipital lobe, and as the cognitive workload increases, the energy of its activity in the parietal lobe gradually weakens. The frontal part is mainly related to human psychological activities. As the difficulty of cognitive workload math problems increases, human psychological activities show obvious fluctuations, indicating that the beta wave is related to people’s psychological functions. The activities of the occipital lobe also explain how beta also has a certain relationship with vision. The 3D EEG topographic map shows that during the entire process, the energy of the beta wave is more active, and it affects the frontal lobe and has a greater ability to monitor the driver’s behaviour.

#### 4.2.5. Trend characteristics analysis of channels occupancy in different wavebands

According to the distribution characteristics of EEG, the changes in the monitor, perception, visual and auditory channels occupied by the four waves under different cognitive workloads were collected, and the results are shown in Table 3.

**Table 3:** Changing trend of the occupancy of each channel under different cognitive workloads.

Wavebands	Workload	Monitor	Perception	Visual	Auditory
Delta	Mild and Moderate	Down	Down	Down	Up
	Moderate and Deep	Up	Down	Down	Up
Theta	Mild and Moderate	Up	Up	Up	Down
	Moderate and Deep	Down	Up	Down	Up
Alpha	Mild and Moderate	Down	Down	Down	Up
	Moderate and Deep	Up	Down	Down	Up
Beta	Mild and Moderate	Up	Up	Up	Down
	Moderate and Deep	Up	Down	Up	Up

Table 3 shows that the changing trend of the delta and alpha waves is almost the same. The monitor channel shows a downtrend with the mild and moderate workload while showing an uptrend with the deep workload. With the increase in cognitive workload, the perception and visual channels show a downward trend, while the auditory channel shows an upward trend.

In the theta wave, the monitor and visual channels all show an upward trend when the workload changes from mild to moderate and decreases with deep workload, while the auditory channel shows the opposite trend. The perception channel always shows an upward trend when the workload deepens.

In the beta wave, the monitor and visual channels all show an upward trend when the cognitive workload deepens. The perception channel shows an upward trend when mild workload changes to moderate and shows a downward trend from moderate to deep workload. The auditory channel shows an opposite trend with the perception channel.

### 4.3. Characteristics analysis of ERSP and ITC of different frequency signals under different levels of cognitive workload

**Figure 9:**
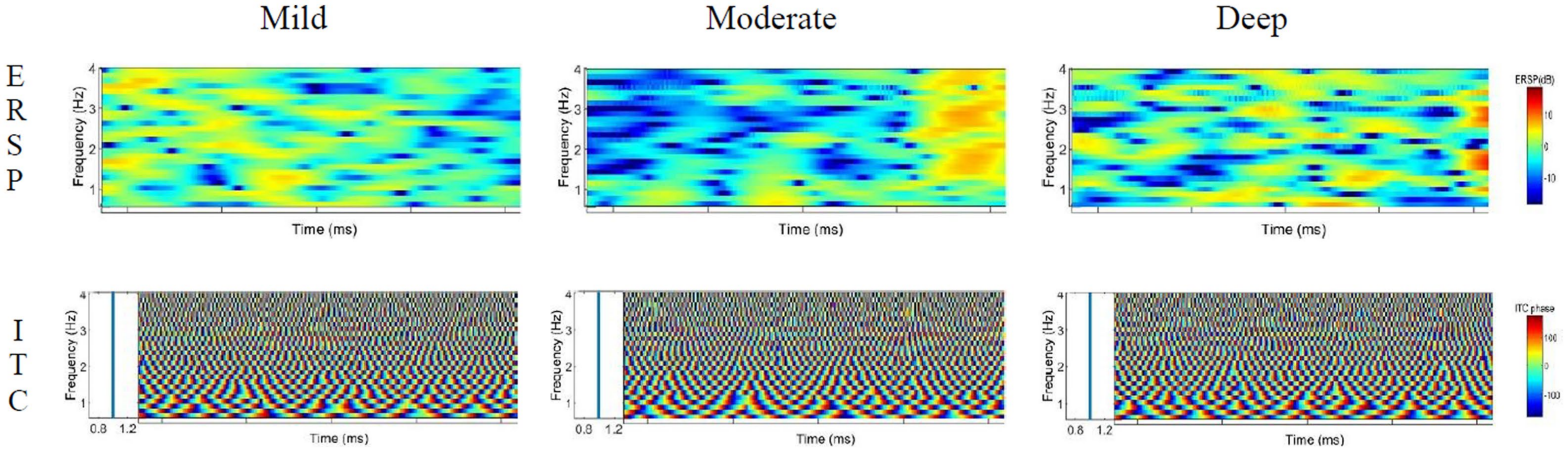
ERSP and ITC characteristics of delta wave under different cognitive workload events.

**Figure 10:**
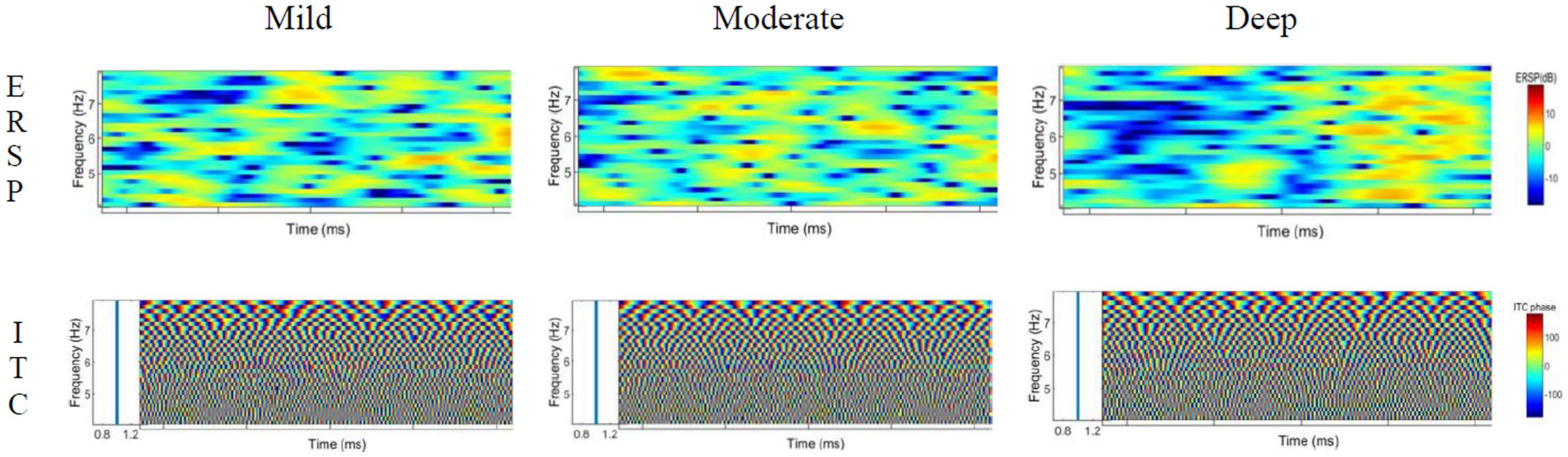
ERSP and ITC characteristics of theta wave under different cognitive workload events.

**Figure 11:**
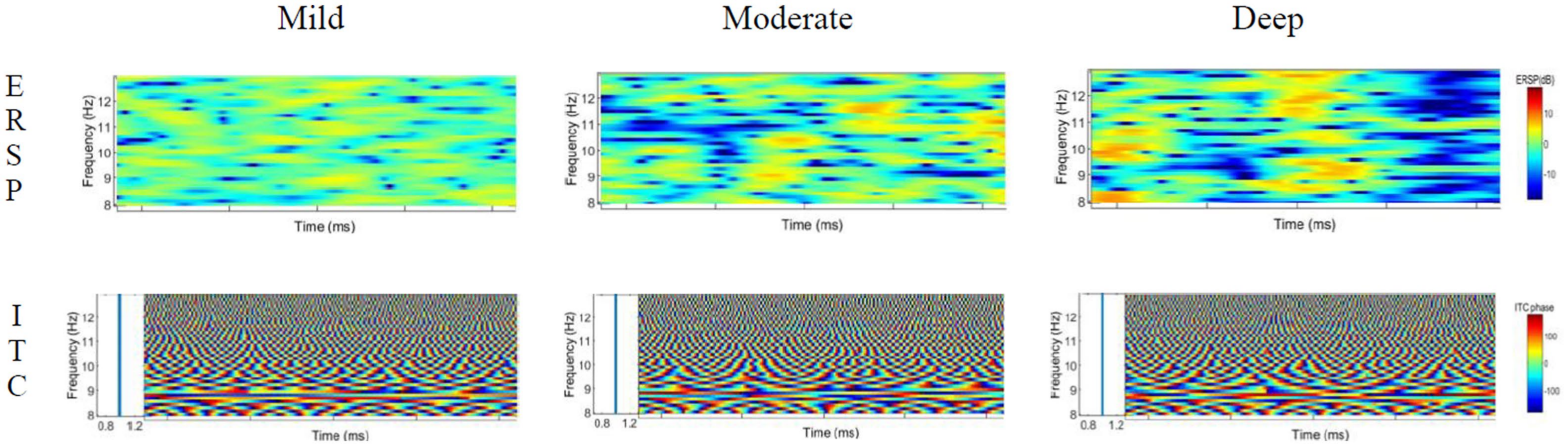
ERSP and ITC characteristics of the alpha wave under different cognitive workload events.

**Figure 12:**
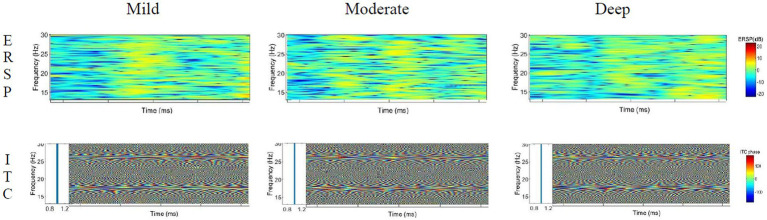
ERSP and ITC characteristics of the beta wave under different cognitive workload events.

The time-frequency distribution diagrams of the ERSP and ITC features of theta, delta, alpha and beta were extracted under different induced distraction workloads, as shown in Figures 9–12. Blue means lower energy, red represents higher energy, and the unit is DB [33].

#### 4.3.1. ERSP and ITC characteristics of delta wave

The analysis of the ERSP characteristics of the delta wave shows that in the process of mild cognitive workload, the energy activity in the front stage is strong, the activity in the rear stage is weak, and the overall energy value is low. During the process of moderate cognitive workload, the delta wave showed a characteristic of energy decline in the early and middle periods, which is an energy-blocking feature, and the energy value was strengthened at the end. In the process of deep cognitive workload, the overall energy activity is relatively uniform, and the activity energy in the last segment is stronger (Especially at 2–3 Hz). The ITC value of the delta wave moves around 1 and is basically 1, indicating that the three levels of cognitive workload processes are all phase-locked.

#### 4.3.2. ERSP and ITC characteristics of theta wave

The analysis of the theta wave ERSP characteristics shows that during the process of mild cognitive workload, the overall energy is weak, the energy at the 7–8 Hz in the middle section is slightly strengthened, and the energy activity at the 5–6 Hz in the latter section is slightly increased. In the process of moderate cognitive workload, the overall activity energy is enhanced compared with that of mild cognitive workload, but it is still weak. In the process of deep cognitive workload, the energy-blocking feature appears at 5–7 Hz in the early stage, and the energy activity was obviously enhanced in the later period. The ITC value of the theta wave is active near 1 and is basically 1, indicating that the three processes are all phase-locked.

#### 4.3.3. ERSP and ITC characteristics of alpha wave

The analysis of the ERSP characteristics of the alpha wave shows that the energy value is relatively stable at different stages in the process of mild cognitive workload. As for the moderate cognitive workload, a brief energy partition at the place of 9–11 Hz in the early stage was observed, and the energy activity afterwards was significantly strengthened. In the process of the deep cognitive workload, an obvious energy blocking phenomenon in the late stage was observed, and the energy activity is more active in the middle stage. The ITC value of the alpha wave moves around 1 and is basically 1, indicating that the three processes are all phase-locked.

#### 4.3.4. ERSP and ITC characteristics of beta wave

The analysis of the ERSP characteristics of the beta wave shows obvious energy barriers during the three distraction workload processes, and the energy activity is strong in the middle of the mild cognitive workload, while in the middle and deep cognitive workload, the energy distribution is relatively uniform, which has been exerting influence on the driver. The ITC value of the beta wave is active near 1, basically 1, indicating that the three distraction processes are all phase-locked.

#### 4.3.5. Changing trend of each channel’s occupancy in different driving states

**Figure 13:**
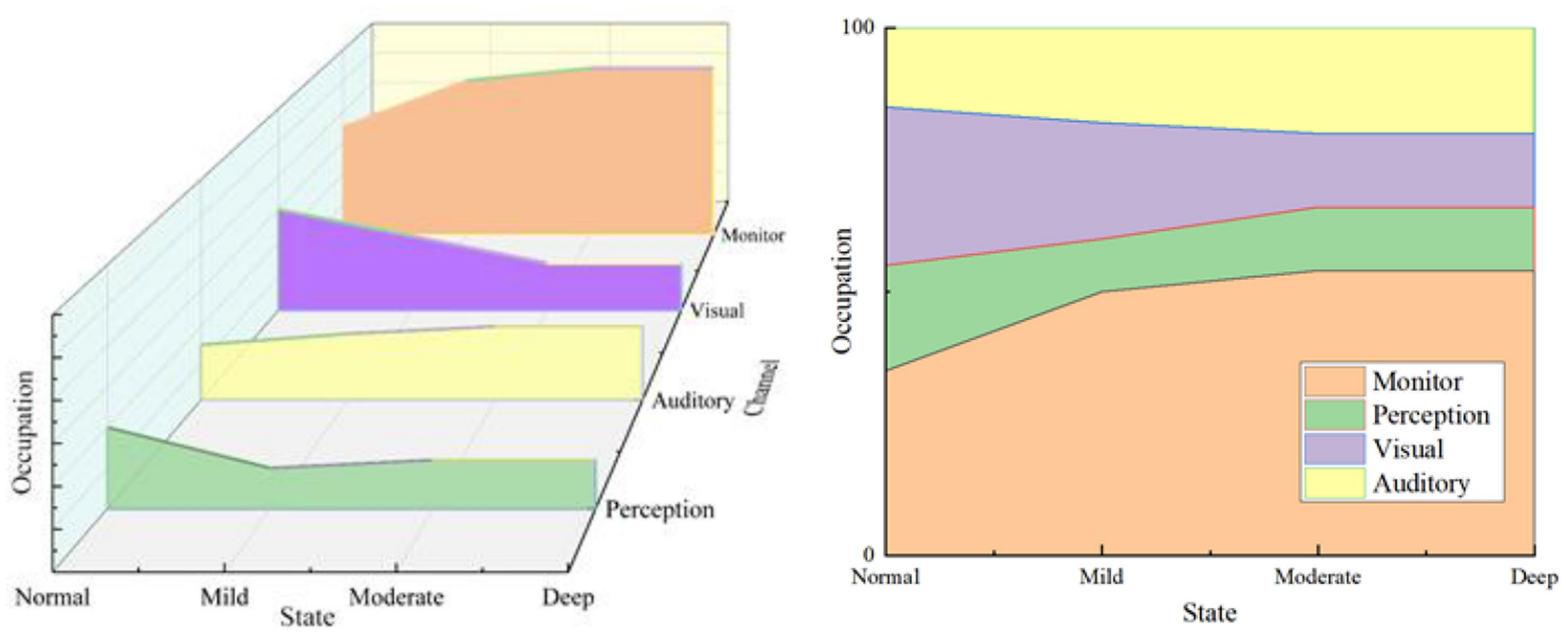
Schematic diagram of the occupancy trend of each channel under different driving states.

According to the ERSP characteristics of each band, combined with the previous research of 4.2.5, the occupation trend of the monitor, perception, visual and auditory channels in different driving conditions can be summarised. The schematic diagram is shown in Figure 13.

The sum of resources occupied by all channels is always 100%. From Figure 13, the resources occupied by the monitor and auditory channel have risen with the increase in cognitive workload, while the resources occupied by the visual channel have been decreasing. The perception channel has shown a slight upward trend in the change from mild to moderate, and the rest of the stage has been in a state of decline.

This result reflects that with the intensification of cognitive workload tasks, the driver’s brain monitoring ability continues to increase to control his behaviour for safe driving. However, due to the progress of cognitive tasks, with the deepening of the degree, the reduction of visual channel resources means that the attention to the external environment is showing a downward trend, but the auditory channel takes up more resources and requires more energy for memory processing. The perception channel resource is also gradually reduced, which also surfaced the intensification of cognitive workload tasks, which has a direct impact on driving safety.

### 4.4. Discussion

With the improvement of brain imaging technology, functional magnetic resonance imaging, functional near-infrared spectroscopy, electroencephalography and magnetoencephalography are important sources to exploring brain activities (Kohlmorgen et al., 2007; Zander and Kothe, 2011; Wang et al., 2014). The cost of memory tasks associated with performing a job in the nervous system appears to be reflected in the magnitude characteristics of EEG task-related regulation. These findings can be interpreted as a human-machine system of the synthesis of mental workload, which are demands related to mental structure placed on the mental resources of users (Gevins et al. (1998)).

The research results of this paper allow us to make the following conclusions:

1. The correlation study of the four brain waves (theta, delta, alpha and beta) under different cognitive states shows that the left and right brain regions perform differently under different degrees of cognitive workload. In terms of the right side, all the different levels of the cognitive workload in all brain waves show significance to normal driving conditions. The left brain showed significant differences except for Theta_Normal – Theta_Mild and Alpha_Normal – Alpha_Moderate. The effect of alpha waves on the right brain is more significant than that on the left.
2. The analysis of EEG topographic maps of different frequency signals shows that the energy activity of the delta wave is relatively weak, which is related to the temporal lobe of the brain to some extent. The theta wave is weak, which is related to the parietal of the brain. The aggravation of cognitive degree will affect human sensory judgment; the energy of alpha and beta waves are more powerful, and they also affect the driver’s ability to monitor behaviour. In addition, the alpha wave mainly affects the occipital region, and the enhancement in cognitive conformity has an impact on the visual ability of humans. The beta wave mainly changes significantly in the frontal part of the brain, indicating that different cognitive workloads have an impact on human psychology. Cooper et al. (2003) shared similar findings to those in this paper that as cognitive workload increases, alpha waves become more intense, but they were based on different cognitive workload tasks. Sonnleitner et al. (2014) also found that when the cognitive workload increased, driving performance decreased, and reaction time and alpha spindle rate increased with working hours. After a deeper cognitive workload, the alpha spindle rate was significantly higher during an auditory secondary (Sonnleitner et al., 2014). Lin et al. conducted a cognitive workload test for the calculation of mathematical problems and did find that theta wave and beta wave in the frontal region of the brain were enhanced after cognitive workload, and the power of the theta wave could represent the degree of interference in normal driving. Savage et al. (2013) found that with increased cognitive workload, θ activity in the frontal lobe increased and θ activity in the occipital lobe decreased. However, the conclusion of this study is based on the electrical interference caused by eye movement. Therefore, the settings of different tasks may have different impacts and need further verification.
3. Analysing the characteristics of ERSP and ITC induced by different frequency signals under different levels of cognitive workload shows that the four brain waves are phase-locked. The energy change of beta waves in each state is relatively uniform, while theta, delta and alpha all showed the phenomenon of energy blocking and sudden energy strengthening under different conditions.
4. As the cognitive workload deepens, the resources occupied by the monitor channel and auditory channel gradually increase, while the resources occupied by the visual and perception channels are on a downward trend. A large amount of cognitive workload is that the driver spends more resources on mind control and memory while the attention to the external environment is weakened, greatly impacting driving safety. Previous studies have shown that activation in the parietal and frontal regions is associated with cognition (Schwarzbach et al., 2002; Kirschen et al., 2005; Zhang et al., 2017). Therefore, a higher task workload may occur, thus occupying cognitive resources.

The research conclusion can improve the driving assistance system to be rationalised to carry out targeted early warning and control of the driver’s workload in different situations.

## 5. Conclusion

This study collects the data of drivers’ brain EEG signal under the normal driving state and different levels of cognitive workload state by conducting an on-road driving test. Based on the collected data, the time-frequency distribution of different EEG signals generated by the left and right brains is studied. The distribution and energy characteristics under different cognitive events are also studied to explore the regular pattern of EEG characteristics of drivers’ brains under different cognitive workloads, as well as the changing trend of monitor, perception and visual and auditory channels in different driving states. Results show that the effect of alpha waves on the right brain is more significant than that on the left, and the four brain waves are phase-locked. As the cognitive workload deepens, the resources occupied by the monitor and auditory channels gradually increase, while the resources occupied by the visual and perception channels are on a downward trend. This paper can provide a theoretical basis for improving driving safety under different cognitive workloads. This paper can also help improve the vehicle driving assistance system and better provide targeted safety warnings for the driver. At the same time, the characteristics of higher levels require more medical knowledge to explore the mysteries of the brain. We will combine more professional medical knowledge to carry out follow-up analysis.

However, this study has certain limitations. First, due to the complexity of implementing the experiments as well as other project constraints (complexity of experiment implementation, funding), we only studied the average characteristics of each state of the sample and do not study the individual heterogeneity in detail in this paper. More participants will be recruited to explore the brain characteristics of drivers under cognitive workload. Gender, occupation and other factors will be screened, and more meaningful factors will be explored. We will also add some driving simulation experiments for supplementary comparative analysis. A comparison of different methods such as texting while driving and N-back should be made with the applied math problem calculation method to find out optimal test results. Moreover, short-time Fourier transform is adopted for time-frequency conversion. Different time windows and different precisions will affect the amplitude and power spectrum of the decomposed signal. Future work will focus on more methods, such as wavelet transform to decompose the signal and verify the different time windows.

## Data availability statement

The raw data supporting the conclusions of this article will be made available by the authors, without undue reservation.

## Author contributions

SQ and RL: conception. RL, GL, and YL: data collection. SH and SQ: formal analysis. GL and RL: software. SH and GL: methodology. SH and RL: funding acquisition. SQ, RL, and YL: writing–original draft. All authors contributed to the article and approved the submitted version.

## Funding

This research was funded by the National Natural Science Foundation of China (grant nos. 52105011 and 52102410), the Young Innovative Talents Project of Guangdong Province (grant nos. 2021KQNCX071 and 2021KQNCX073), the Tertiary Education Scientific Research Project of Guangzhou Municipal Education Bureau (grand no. 202235334), the Basic and Applied Basic Research Projects of Guangzhou (grand nos. SL2023A04J00685 and SL2023A04J00686).

## Conflict of interest

The authors declare that the research was conducted in the absence of any commercial or financial relationships that could be construed as a potential conflict of interest.

## Publisher’s note

All claims expressed in this article are solely those of the authors and do not necessarily represent those of their affiliated organizations, or those of the publisher, the editors and the reviewers. Any product that may be evaluated in this article, or claim that may be made by its manufacturer, is not guaranteed or endorsed by the publisher.
